# Supplementary material for: Lactate Promotes Hypoxic Granulosa Cells’ Autophagy by Activating the HIF-1α/BNIP3/Beclin-1 Signaling Axis
Source: Genes (Basel). 2024 Dec 26;16(1):14. doi: 10.3390/genes16010014 (PMC11765430; doi:10.3390/genes16010014)
Supplement: Supplementary file 1 [file genes-16-00014-s001.zip › Supplementary materials.pdf]

Lactate Promotes Hypoxic Granulosa Cells' Autophagy by Activating the HIF-1α/BNIP3/Beclin-1 Signaling Axis

Yitong Pan, Gang Wu, Min Chen, Xiumei Lu, Ming Shen, Hongmin Li and Honglin Liu *

College of Animal Science and Technology, Nanjing Agricultural University, Nanjing 210095, China
* Correspondence: liuhonglin@njau.edu.cn; Tel./Fax: +86-25-84395106

Supplementary Table S1. siRNA sequences

Scrambled siRNA	Sense (5'-3')	UUCUCCGAACGUGUCACGUTT
	Antisense (5'-3')	ACGUGACACGUUCGGAGAATT
LDHA-579	Sense (5'-3')	CGGUUGCAAUCUGGAUUCATT
	Antisense (5'-3')	UGAAUCCAGAUUGCAACCGTT
LDHB-424	Sense (5'-3')	CUGGUGCAGAGAAAUGUUATT
	Antisense (5'-3')	UAACAUUUCUCUGCACCAGTT
HIF-1-α-960	Sense (5'-3')	GCCGCUCAAUUUAUGAAUATT
	Antisense (5'-3')	UAUUCAUAAAUUGAGCGGCTT
BNIP3	Sense (5'-3')	ACGGAAGGAAGACUUUGAGTT
	Antisense (5'-3')	CUCAAAGUCUUCCUUCCGUTT

Supplementary Figure S1 Knockdown of LDHA/B inhibits hypoxia-induced autophagy in granulosa cells

(A) Cells were cultured under normoxic (21% O_2) or hypoxic (1% O_2) conditions for 12 h, and intracellular LC3 and p62 protein levels were analyzed by western blot. (B) Quantitative analysis of LC3-I to LC3-II conversion. Data represent mean ± SD; $n \geq 3$; $^{**}P < 0.01$. (C) Quantitative analysis of p62 protein levels. Data represent mean ± SD; $n \geq 3$; $^{*}P < 0.05$. (D) Immunofluorescence localization of GFP-LC3 in granulosa cells. (E) Quantification of GFP-LC3 puncta per cell. Data represent mean ± SD; $^{**}P < 0.01$. At least 5 cells per group were counted. (F) Cellular lactate levels were measured after silencing or non-silencing of LDHA under hypoxic or normoxic conditions. Data represent mean ± SD; $^{****}P < 0.0001$. (G) Western blot analysis of LDHA protein levels in cells transfected with LDHA siRNA or scrambled siRNA for 24 h. (H) Western blot analysis of LDHB protein levels in cells transfected with LDHB siRNA or scrambled siRNA for 24 h. (I) Quantitative analysis of LDHA protein levels. Data represent mean ± SD; $n \geq 3$; $^{***}P < 0.001$. (J) Quantitative analysis of LDHB protein levels. Data represent mean ± SD; $n \geq 3$; $^{**}P < 0.01$. (K) Cells were divided into LDHA and LDHB silencing and non-silencing groups using siRNA, cultured under normoxic or hypoxic conditions for 12 h, and intracellular LC3 and p62 protein levels were detected by western blot. (L) Quantitative analysis of LC3-I to LC3-II conversion. Data represent mean ± SD; $n \geq 3$; $^{****}P < 0.0001$. (M) Quantitative analysis of p62 protein levels. Data represent mean ± SD; $n \geq 3$; $^{**}P < 0.01$; $^{*}P < 0.05$. (N) Cellular lactate levels were measured in LDHA and LDHB silencing and non-silencing groups under hypoxic or normoxic conditions. Data represent mean ± SD; $n \geq 3$; $^{*}P < 0.05$. Error bars represent the standard deviation of the mean.

Supplementary Figure S2 Inhibition of HIF-1α suppresses lactate-induced autophagy in granulosa cells under hypoxia

(A) Cells were transfected with HIF-1α siRNA or scrambled siRNA for 24 h, and HIF-1α protein levels were detected by western blot. (B) Quantitative analysis of HIF-1α protein levels. Data represent mean ± SD; $n \geq 3$; ****$P < 0.0001$. (C) Cells were treated with PX478 to inhibit HIF-1α and then cultured under normoxic or hypoxic conditions. Protein levels of LC3, p62, Beclin-1, and BNIP3 were analyzed by western blot. (D) Quantitative analysis of LC3-I to LC3-II conversion. Data represent mean ± SD; $n \geq 3$; ****$P < 0.0001$; *$P < 0.05$. (E) Quantitative analysis of Beclin-1, BNIP3, and p62 protein levels. Data represent mean ± SD; $n \geq 3$; ****$P < 0.0001$; ***$P < 0.001$; **$P < 0.01$; *$P < 0.05$. (F) Cells were treated with PX478 to inhibit HIF-1α for 12 h, followed by the addition of 15 mM Nala and further incubation for 12 h. Protein levels of LC3, p62, Beclin-1, and BNIP3 were detected by western blot. (G) Quantitative analysis of LC3-I to LC3-II conversion. Data represent mean ± SD; $n \geq 3$; ***$P < 0.001$; *$P < 0.05$. (H) Quantitative analysis of Beclin-1, BNIP3, and p62 protein levels. Data represent mean ± SD; $n \geq 3$;****$P < 0.0001$; ***$P < 0.001$; **$P < 0.01$; *$P < 0.05$. (I) Cells were transfected with BNIP3 siRNA or scrambled siRNA for 24 h, and BNIP3 protein levels were detected by western blot. (J) Quantitative analysis of BNIP3 protein levels. Data represent mean ± SD; $n \geq 3$; ***$P < 0.001$. Error bars represent the standard deviation of the mean.
